# Quantitative proteomics analysis to identify biomarkers of refractory benign paroxysmal positional vertigo

**DOI:** 10.1016/j.bjorl.2026.101771

**Published:** 2026-02-05

**Authors:** Liang Xia, Kexin Song, Lili Xiao, Yi Chen, Hui Li, Yanmei Feng

**Affiliations:** a Department of Otolaryngology-Head and Neck Surgery, Shanghai Sixth People’s Hospital Affiliated to Shanghai Jiao Tong University School of Medicine, Shanghai, China; b Department of Ophthalmology, Shanghai Sixth People’s Hospital Affiliated to Shanghai Jiao Tong University School of Medicine, Shanghai, China

**Keywords:** Benign paroxysmal positional vertigo, Biomarker, Mass spectrometry, Peptide, Tandem mass tag

## Abstract

**Objective:**

Benign Paroxysmal Positional Vertigo (BPPV) is transient vertigo and paroxysmal nystagmus induced by changes in head position. This study was conducted to investigate the differential expression of serum proteins in patients with refractory BPPV and to screen for diagnostic biomarkers.

**Methods:**

Serum samples were collected from patients with BPPV; tandem mass tag-based quantitative proteomics technology was used to detect and quantify the serum proteins of 30 individuals with refractory BPPV and 30 control volunteers. Bioinformatics analysis of differentially expressed proteins was performed using hierarchical clustering, gene ontology annotation, Kyoto Encyclopedia of Genes and Genomes analysis, and protein-protein interaction network analysis.

**Results:**

A total of 769 proteins were identified, and 57 differentially expressed proteins were screened between the two groups; 15 proteins were upregulated, whereas 42 were downregulated. Five differentially expressed proteins were chosen for parallel reaction monitoring analysis to confirm the results. Apolipoprotein A-I, apolipoprotein A-II, apolipoprotein C-III, fibrinogen gamma chain, and fructose-bisphosphate aldolase B were the five potential candidate biomarker proteins.

**Conclusions:**

This is the first quantitative proteomic study to reveal diagnostic biomarkers in patients with BPPV using tandem mass tag labeling technology. The identified differential proteins may improve the understanding of the pathogenesis and molecular mechanism of BPPV.

**Level of evidence:**

Level 4.

## Highlights

• This is the first TMT-based proteomics study on refractory BPPV.
• 57 potential biomarkers identified significant for improving diagnostic accuracy.
• BPPV mechanisms are related to cholesterol metabolism and platelet activation.

## Introduction

Benign Paroxysmal Positional Vertigo (BPPV) is defined as transient vertigo and paroxysmal nystagmus induced by changes in the head position. It is the most common peripheral vestibular disease.^1^ 5.6 million outpatients complain of dizziness in the United States each year, and between 17% and 42% of patients with vertigo are diagnosed with BPPV.^2^ Two basic theories, cupulolithiasis and canalolithiasis, were considered to be involved in the pathophysiology of BPPV.^3^^,^^4^ BPPV is caused by abnormal stimulation of the cupula. When the head position changes, otoliths either float or attach to the cupula in any of the three semicircular canals. BPPV is typically diagnosed by the Dix-Hallpike maneuver and supine rotation test and treated by specific canalith repositioning maneuvers. Most patients can be cured by this treatment. Even in the absence of treatment, patients can recover within a few days.^5^ Although most patients with BPPV have a good prognosis, there are occasional severe refractory cases that can be debilitating to patients and severely decrease their quality of life.^6^

The specific etiological factor is detected in only a minority of patients; therefore, the etiology of BPPV remains unknown. The main causes of secondary BPPV may be Meniere’s disease (0.5%–30%), head injury (8.5%–27%), vestibular neuritis (0.8%–20%), and sensorineural hearing loss (0.2%–5%).^7^, ^8^, ^9^, ^10^, ^11^, ^12^ Recent clinical evidence further indicates that asymmetric hearing loss significantly predicts ipsilateral otoconial displacement, with 70% of BPPV episodes occurring in the worst-hearing ear.^13^ Additionally, sarcoidosis, myocardial infarction, carcinomas treated by chemotherapy and/or radiotherapy, leukemia, and active ulcerative colitis have been reported as less frequent causes of BPPV.^14^ Furthermore, a few studies have identified metabolic changes as sources of secondary BPPV.^15^ Therefore, the specific molecular mechanism of BPPV remains unclear. Diagnosis is still based on clinical examination, and no specific protein or biomarker has been identified.

Given the multifactorial pathophysiology of BPPV, we hypothesize that specific molecular alterations occur systemically and may be detectable in the peripheral blood. Mass Spectrometry (MS)-based serum proteomics offers a high-throughput, sensitive, and unbiased platform for the comprehensive profiling of proteins, enabling the identification of potential disease-associated biomarkers.^16^ Serum is a readily accessible biofluid that reflects both systemic and, potentially, inner-ear-related pathophysiological changes, making it an attractive matrix for biomarker discovery in vestibular disorders. Previous studies have successfully employed MS-based proteomics to investigate other vestibular-related conditions, such as Ménière’s disease and vestibular schwannoma, identifying candidate proteins associated with disease mechanisms and progression.^17^^,^^18^ Building upon these precedents, we applied Tandem Mass Tag (TMT) in quantitative proteomics analysis to evaluate differences in serum protein expression between a healthy control group and refractory BPPV group. Simultaneously, five interesting proteins were selected for Parallel Reaction Monitoring (PRM) analysis to confirm the MS results. Advances in proteomics ensure the accuracy and effectiveness of these studies.

Therefore, this study evaluated differentially expressed serum proteins in patients with refractory BPPV using a proteomics-driven approach and identified potential biomarkers for the potential etiologic implications of refractory BPPV at the molecular level.

## Methods

### Subjects and sample collection

From September 2017 to December 2018, we recruited 363 patients with BPPV. BPPV diagnosis was made based on the criteria described by the Bárány Society.^19^ Patients with head trauma, history of surgery, history of otology diseases, migraine, and history of cervical spondylosis were excluded. Patients with systemic diseases such as renal insufficiency, hepatic illnesses, thyroid disorders, and hyperlipidemia were also excluded. All patients were treated with canalith repositioning maneuvers once per week. A total of 18 patients were excluded due to being lost to follow-up. After one month of treatment, 30 patients showed no improvement or worsening in their vertigo symptoms or positional nystagmus. These patients were considered to have refractory BPPV and were included in the refractory group. Simultaneously, serum samples of 30 healthy volunteers (control group) were collected during routine medical check-up. We also excluded systemic diseases such as renal insufficiency, hepatic illnesses, thyroid disorders, and hyperlipidemias in the control group. The clinical characteristics of the subjects are shown in Table 1.

**Table 1:** Participant characteristics between the refractory group and control group.

	Refractory group (n = 30)			Control group (n = 30)			p-value
Sample replicates	Nº1	Nº2	Nº3	Nº4	Nº5	Nº6	
Age (Mean ± SD)	43.10 ± 8.73	43.1 ± 12.39	45.90 ± 10.36	40.80 ± 3.61	39.40 ± 2.54	42.20 ± 6.95	>0.05
Man (n)	4	6	5	5	5	5	>0.05
Women (n)	6	4	5	5	5	5	>0.05

**Fig. 1:**
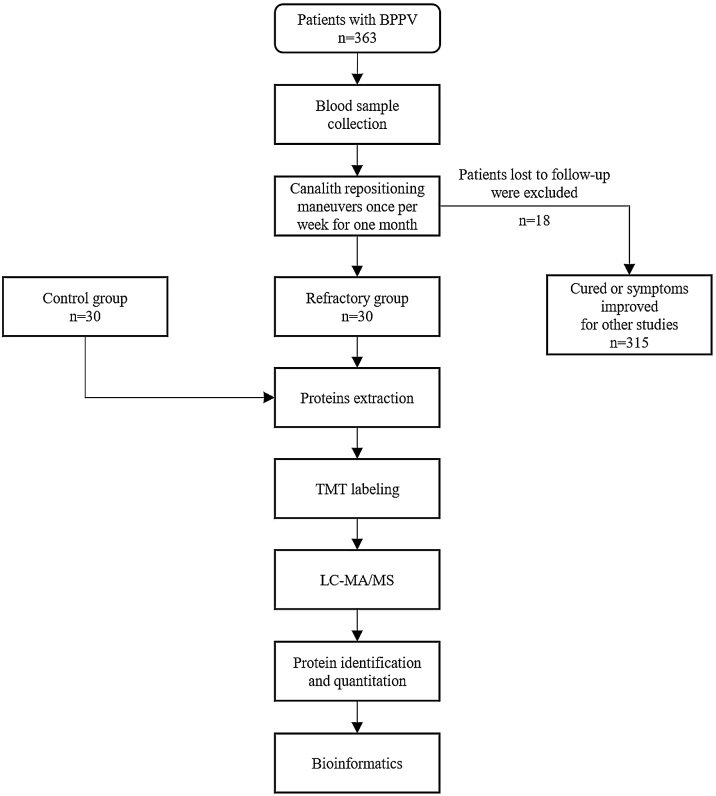
Experiment design. Flowchart of the enrollment process and experimental procedures.

In the refractory group, blood samples were collected before treatment. After centrifugation, the serum was stored at −80 °C. In the control group, serum from 30 subjects was divided into three parts, numbered as 1, 2, and 3. In the refractory group, the serum from 30 subjects was divided into three parts, numbered as 4, 5, and 6. The flowchart of the study process is shown in Fig. 1.

### Sample preparation

Abundant proteins in the serum pools were depleted using an Agilent Multiple Affinity Removal Column (Agilent Technologies, Santa Clara, CA, USA). Low-abundance components were desalted and concentrated using an ultrafiltration tube (Sartorius, Göttingen, Germany). The 5× loading buffer was mixed with 20 μg of protein, boiled for 5-min, and separated by 12.5% (sodium dodecyl sulfate ‒ polyacrylamide gel electrophoresis) SDS-PAGE. Protein bands were detected by Coomassie Blue R-250 staining.

### Filter-aided sample preparation

Protein samples (200 μg) were mixed with 30 μL SDT Buffer (Sodium Deoxycholate/Tris-based lysis buffer) for each sample. The low-molecular weight components were removed through repeated ultrafiltration. Thereafter, each sample was incubated in the dark for 30 min and digested by trypsin (Promega, Madison, WI, USA). The resulting peptide was collected and estimated by measuring the UV absorbance at 280 nm.

### TMT labeling and MS

Each sample was labeled by TMT reagent according to the manufacturer’s protocol (Thermo Fisher Scientific, Waltham, MA, USA). A Pierce high pH reversed-phase peptide fractionation kit was used to fractionate the TMT-labeled digest of each sample into 10 fractions. The fractions were subjected to nano Liquid Chromatography Coupled to tandem Mass Spectrometry (LC-MS/MS) analysis. The peptide compound was loaded onto a reverse-phase trap column linked to a C18-reversed phase analytical column in buffer A (0.1% formic acid) and detached with a rectilinear gradient of buffer B (84% acetonitrile and 0.1% formic acid). LC-MS/MS analysis was performed on a Q Exactive mass spectrometer (Thermo Fisher Scientific). Finally, spectra were detected by MASCOT engine (Matrix Science, London, UK; version 2.2) in Proteome Discoverer 1.4.

### Hierarchical clustering

Hierarchical clustering analysis was performed using the relative expression data of the studied protein. For this, Cluster 3.0 (http://bonsai.hgc.jp/∼mdehoon/software/cluster/software.htm) and Java TreeView software (http://jtreeview.sourceforge.net) were used.

### Gene Ontology (GO) and Kyoto Encyclopedia of Genes and Genomes (KEGG) pathway annotation

The differentially expressed proteins were examined with the UniProtKB database (Release 2016_10) and retrieved sequences were scanned in the SwissProt database using NCBI BLAST + client software (NCBI blast 2.2.28+ win32.exe) to identify homologous sequences. To further explore the role of differentially expressed proteins in the physiological process of cells, enrichment analysis was performed. GO enrichment of three terms (biological process, molecular function, and cellular component) and KEGG pathway enrichment were performed based on the Fisher’s exact test. Benjamini-Hochberg correction for multiple experiments was further applied to adjust the derived p-value.

### Protein-Protein Interaction (PPI) network analysis

The PPI information of different proteins was searched in IntAct molecular interaction database (http://www.ebi.ac.uk/univate/) using gene symbols or STRING software (http://STRINGdb.org/). The results were imported into Cytoscape software (http://www.Cytoscape.org/, version 3.2.1) to visualize and analyze the functional PPI networks. Protein gradation was calculated to assess its rank in the PPI network.

### PRM analysis

The peptide was isolated using the Easy-Nlc 1200 (Thermo Fisher Scientific). MS analysis was performed with a Q-Exactive HF mass spectrometer (Thermo Fisher Scientific) in PRM mode. Skyline 3.5.0 software was used for PRM data analysis.

## Results

### LC-MS/MS analysis

**Fig. 2:**
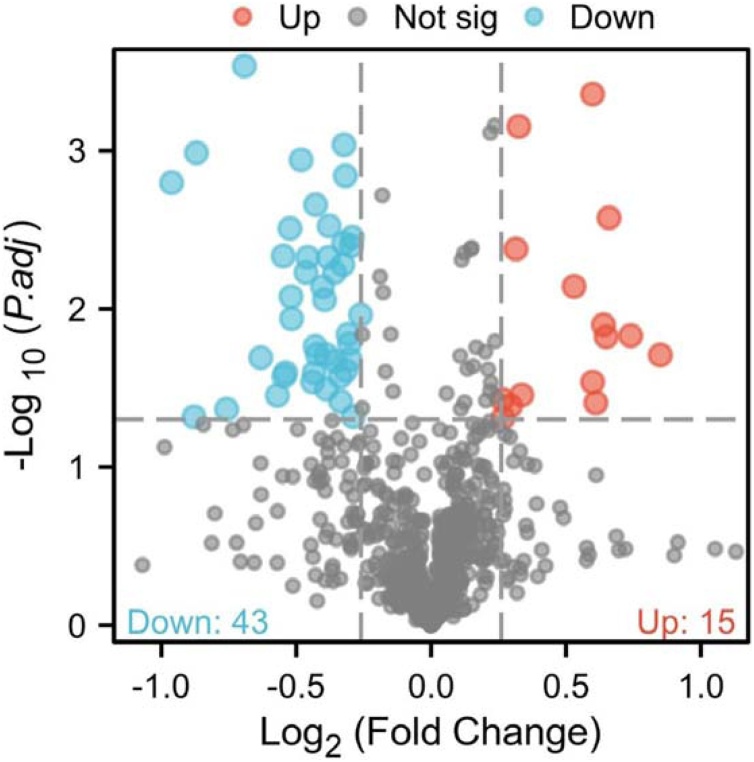
Volcano plot. Volcano plot was prepared using the two factors of protein expression difference (fold-change) and p-value obtained by *t*-test between the two groups of samples. The abscissa is the difference multiple (logarithm change with 2 as the base), ordinate is the significant p-value of the difference (logarithm change with the base as 10). Red and Blue dots in the figure are significant differentially expressed proteins (fold-change greater than 1.2 and p < 0.05), and grey dots are proteins with no significant change.

Samples from invalid group (refractory BPPV) patients and control group patients were labeled and then differential proteomic screening was performed (Fig. 1). A total of 769 corresponding proteins were identified by MS. Differentially expressed proteins were selected as those showing a fold-change in expression of more than 1.2-fold (up-regulation greater than 1.2-fold or down-regulation 0.83-fold) and p-value < 0.05. Fifty-seven differentially expressed proteins (15 increased and 42 decreased) were identified between the refractory BPPV and control groups (Table 2). The quantitative results of protein expression in the two groups are shown in a volcano plot (Fig. 2).

**Table 2:** Differentially expressed proteins between groups.

Accession	Protein Name	Regulated type	Gene Name	Refractory/Control	p-value
P02679	Fibrinogen gamma chain	Up	FGG	1.802292	0.019606
P02675	Fibrinogen beta chain	Up	FGB	1.670026	0.014794
Q15166	Serum paraoxonase / Lactonase 3	Up	PON3	1.579738	0.002653
P02656	Apolipoprotein C-III	Up	APOC3	1.56808	0.015044
P00488	Coagulation factor XIII A chain	Up	F13A1	1.557187	0.012619
P05062	Fructose-bisphosphate aldolase B	Up	ALDOB	1.527011	0.039289
P02655	Apolipoprotein C-II	Up	APOC2	1.514481	0.029122
P27169	Serum paraoxonase / Arylesterase 1	Up	PON1	1.514283	0.000438
P02652	Apolipoprotein A-II	Up	APOA2	1.443836	0.007214
P27930	Interleukin-1 receptor type 2	Up	IL1R2	1.262427	0.035121
P05090	Apolipoprotein D	Up	APOD	1.252904	0.000701
Q16610	Extracellular matrix protein 1	Up	ECM1	1.243737	0.004183
P49720	Proteasome subunit beta type-3	Up	PSMB3	1.226395	0.04118
P01008	Antithrombin-III	Up	SERPINC1	1.202868	0.049039
P78417	Glutathione S-transferase omega-1	Up	GSTO1	1.200654	0.037549
P52566	Rho GDP-dissociation inhibitor 2	Down	ARHGDIB	0.818814	0.048147
P01825	Immunoglobulin heavy variable 4−59	Down	IGHV4-59	0.816536	0.003526
A0A0B4J1V0	Immunoglobulin heavy variable 3−15	Down	IGHV3-15	0.814956	0.003999
P0DOX6	Immunoglobulin mu heavy chain	Down	–	0.811919	0.020846
P02788	Lactotransferrin	Down	LTF	0.810589	0.016632
P40197	Platelet glycoprotein V	Down	GP5	0.807806	0.014511
P16109	P-selectin	Down	SELP	0.803161	0.0245
P07359	Platelet glycoprotein Ib alpha chain	Down	GP1BA	0.80221	0.001438
P49767	Vascular endothelial growth factor C	Down	VEGFC	0.799429	0.000914
P09486	SPARC	Down	SPARC	0.798568	0.003837
P07737	Profilin-1	Down	PFN1	0.797026	0.005261
A0A087WSY6	Immunoglobulin kappa variable 3D-15	Down	IGKV3D-15	0.792598	0.038455
P01714	Immunoglobulin lambda variable 3−19	Down	IGLV3-19	0.79156	0.02691
A0A0B4J1U7	Immunoglobulin heavy variable 6−1	Down	IGHV6-1	0.780852	0.021403
P0DOY2	Immunoglobulin lambda constant 2	Down	IGLC2	0.780249	0.005884
P05121	Plasminogen activator inhibitor 1	Down	SERPINE1	0.769499	0.002989
P10599	Thioredoxin	Down	TXN	0.767911	0.004733
P02647	Apolipoprotein A-I	Down	APOA1	0.764293	0.032257
Q13201	Multimerin-1	Down	MMRN1	0.763762	0.019549
P23284	Peptidyl-prolyl cis-trans isomerase B	Down	PPIB	0.760497	0.008765
P02775	Platelet basic protein	Down	PPBP	0.756569	0.007239
P69905	Hemoglobin subunit alpha	Down	HBA1	0.746545	0.019405
P27105	Erythrocyte band 7 integral membrane protein	Down	STOM	0.743248	0.002194
P62937	Peptidyl-prolyl cis-trans isomerase A	Down	PPIA	0.742432	0.017276
O00151	PDZ and LIM domain protein 1	Down	PDLIM1	0.740681	0.025357
Q9P1F3	Costars family protein ABRACL	Down	ABRACL	0.73747	0.029036
P05067	Amyloid-beta precursor protein	Down	APP	0.728226	0.004724
P01709	Immunoglobulin lambda variable 2−8	Down	IGLV2-8	0.725195	0.005888
P01871	Immunoglobulin heavy constant mu	Down	IGHM	0.71603	0.001141
A0A0C4DH29	Immunoglobulin heavy variable 1−3	Down	IGHV1-3	0.698684	0.011537
A0A075B6P5	Immunoglobulin kappa variable 2−28	Down	IGKV2-28	0.697799	0.008382
P0DP03	Immunoglobulin heavy variable 3-30-5	Down	IGHV3-30-5	0.695801	0.003096
P37802	Transgelin-2	Down	TAGLN2	0.688071	0.025361
P68871	Hemoglobin subunit beta	Down	HBB	0.685268	0.026741
P0DOX2	Immunoglobulin alpha-2 heavy chain	Down	–	0.68384	0.00463
Q86YW5	Trem-like transcript 1 protein	Down	TREML1	0.673723	0.035329
P02776	Platelet factor 4	Down	PF4	0.64554	0.020381
P10124	Serglycin	Down	SRGN	0.618958	0.00029
P02787	Serotransferrin	Down	TF	0.591389	0.043176
P07996	Thrombospondin-1	Down	THBS1	0.547137	0.001029
P62328	Thymosin beta-4	Down	TMSB4X	0.543741	0.048269
Q9HCN6	Platelet glycoprotein VI	Down	GP6	0.513295	0.00159

### Hierarchical clustering analysis

**Fig. 3:**
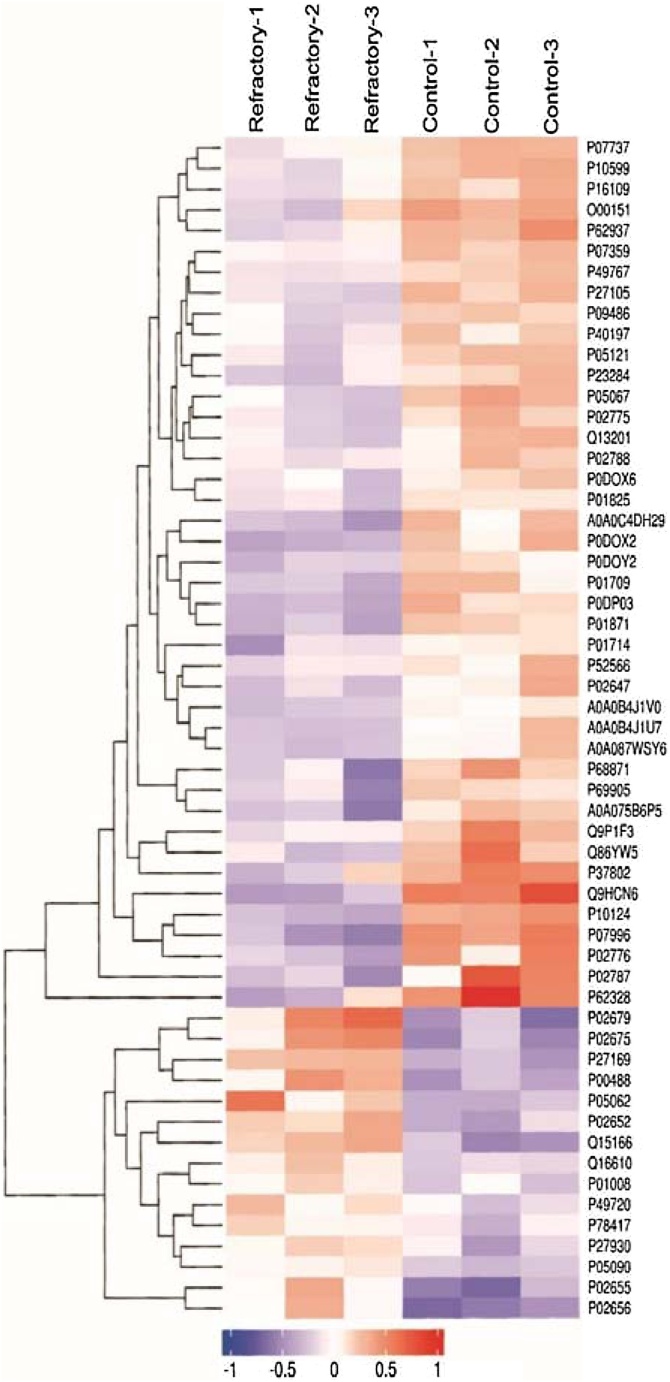
Hierarchical cluster. Significant differentially expressed proteins between the two groups were well-distinguished by hierarchical clustering analysis. Hierarchical clustering of changes in abundance of differentially expressed proteins. Horizontal comparison was performed to classify samples into three categories, suggesting that the selected differentially expressed proteins effectively distinguished between samples. Vertical comparison indicated the proteins as classifiable into two categories with opposite directional variation, demonstrating the rationality of the selected differentially expressed proteins.

The results of hierarchical clustering are shown in Fig. 3. The log_2_-expression of differentially expressed proteins is displayed in different colors in the heat map. A red square indicates upregulation and green square represents downregulation. The differentially expressed proteins screened in this study effectively distinguished the invalid and control groups and validated the identified differentially expressed proteins.

### GO functional annotation analysis

**Fig. 4:**
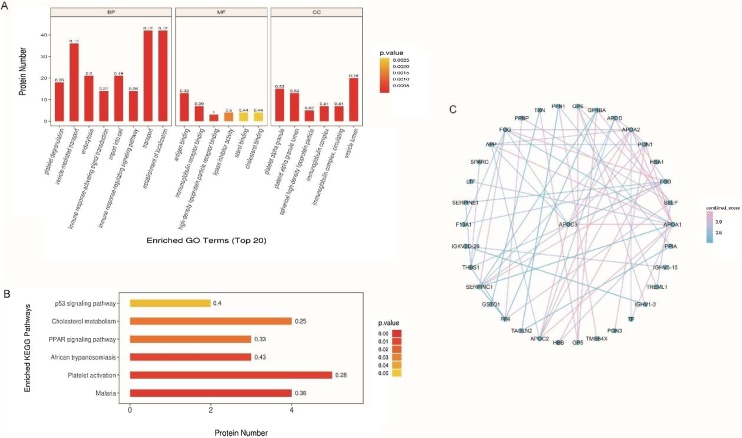
GO annotation, KEGG pathway analysis and PPI network. (A) GO annotation results of differentially expressed proteins. Abscissa in the figure represents the GO level 2 explanatory information, including biological process, molecular function, and cellular component. The left ordinate represents the number of differentially expressed proteins under each functional classification and right ordinate represents the percentage of differentially expressed proteins under each functional classification of the total number of differentially expressed proteins. (B) KEGG pathway analysis of significantly altered pathways. Each number above the bar charts is the Rich factor (≤1), given by the ratio of the number of proteins annotated in each category. The results show that platelet activation, PPAR signaling pathway, cholesterol metabolism, and p53 signaling pathways were changed significantly. (C) PPI network of significant differentially expressed proteins. Blue nodes represent proteins, and lines represent protein-protein interactions.

In high-throughput proteomics, is important to understand which functions are greatly influenced by biological processing. We used Blast2GO (https://www.Blast2Go.com/) software for Gene Ontology (GO) analysis of the screened differentially expressed proteins. According to the second level (level 2) results, the differentially expressed proteins mainly participated in cellular process, biological regulation, response to stimulus, regulation of biological process, and metabolic process. The proteins may also have some catalytic, regulatory, signal transducer, or molecular transducer activities (Fig. 4A).

### KEGG pathway enrichment

Proteins interact with each other to perform certain biochemical reactions and exert biological functions. Though KEGG pathway analysis, we identified 63 enriched KEGG pathways (Table 3). KEGG pathway enrichment indicated significant changes in platelet activation, the PPAR signaling pathway, cholesterol metabolism, and p53 signaling pathways (Fig. 4B). The horizontal axis of the graph represents the number of significant proteins, whereas the vertical axis shows the enriched KEGG pathways. The color of the bar graph represents the significance of the enriched KEGG pathways. Colors closer to red color indicate a lower p-value and more significant enrichment. The number on the bar corresponds to the Rich factor (≤1), which is the ratio of the number of marked proteins in each category.

**Table 3:** KEGG pathway analysis of the differentially expressed proteins.

Map_ID	Map_Name	Seqs num	Seq
hsa05144	Malaria	4	P16109 P69905 P68871 P07996
hsa04611	Platelet activation	5	P02679 P02675 P40197 P07359 Q9HCN6
hsa05143	African trypanosomiasis	3	P02647 P69905 P68871
hsa03320	PPAR signaling pathway	3	P02656 P02652 P02647
hsa04979	Cholesterol metabolism	4	P02656 P02655 P02652 P02647
hsa04115	p53 signaling pathway	2	P05121 P07996
hsa04640	Hematopoietic cell lineage	3	P27930 P40197 P07359
hsa05204	Chemical carcinogenesis	1	P78417
hsa04962	Vasopressin-regulated water reabsorption	1	P52566
hsa00982	Drug metabolism - cytochrome P450	1	P78417
hsa00980	Metabolism of xenobiotics by cytochrome P450	1	P78417
hsa04062	Chemokine signaling pathway	2	P02775 P02776
hsa04060	Cytokine-cytokine receptor interaction	3	P27930 P02775 P02776
hsa04512	ECM-receptor interaction	4	P40197 P07359 P07996 Q9HCN6
hsa05418	Fluid shear stress and atherosclerosis	3	P27930 P78417 P10599
hsa04726	Serotonergic synapse	1	P05067
hsa04217	Necroptosis	1	P62937
hsa04978	Mineral absorption	1	P02787
hsa01524	Platinum drug resistance	1	P78417
hsa04933	AGE-RAGE signaling pathway in diabetic complications	2	P49767 P05121
hsa04977	Vitamin digestion and absorption	1	P02647
hsa04975	Fat digestion and absorption	1	P02647
hsa04015	Rap1 signaling pathway	3	P49767 P07737 P07996
hsa03050	Proteasome	1	P49720
hsa00983	Drug metabolism - other enzymes	1	P78417
hsa04066	HIF-1 signaling pathway	2	P05121 P02787
hsa00051	Fructose and mannose metabolism	1	P05062
hsa04621	NOD-like receptor signaling pathway	1	P10599
hsa05219	Bladder cancer	1	P07996
hsa04722	Neurotrophin signaling pathway	1	P52566
hsa04668	TNF signaling pathway	1	P49767
hsa04610	Complement and coagulation cascades	5	P02679 P02675 P00488 P01008 P05121
hsa05215	Prostate cancer	1	P27930
hsa05225	Hepatocellular carcinoma	1	P78417
hsa04218	Cellular senescence	1	P05121
hsa04390	Hippo signaling pathway	1	P05121
hsa04926	Relaxin signaling pathway	1	P49767
hsa04216	Ferroptosis	1	P02787
hsa05202	Transcriptional misregulation in cancer	1	P27930
hsa04371	Apelin signaling pathway	1	P05121
hsa00030	Pentose phosphate pathway	1	P05062
hsa00480	Glutathione metabolism	1	P78417
hsa05010	Alzheimer disease	1	P05067
hsa05166	Human T-cell leukemia virus 1 infection	1	P27930
hsa04350	TGF-beta signaling pathway	1	P07996
hsa05142	Chagas disease (American trypanosomiasis)	1	P05121
hsa05131	Shigellosis	1	P07737
hsa04810	Regulation of actin cytoskeleton	2	P07737 P62328
hsa05150	Staphylococcus aureus infection	2	P02679 P16109
hsa05132	Salmonella infection	1	P07737
hsa00010	Glycolysis / Gluconeogenesis	1	P05062
hsa05200	Pathways in cancer	2	P78417 P49767
hsa05206	MicroRNAs in cancer	1	P07996
hsa04014	Ras signaling pathway	1	P49767
hsa05146	Amoebiasis	1	P27930
hsa04510	Focal adhesion	2	P49767 P07996
hsa04151	PI3K-Akt signaling pathway	2	P49767 P07996
hsa04010	MAPK signaling pathway	1	P49767
hsa05165	Human papillomavirus infection	1	P07996
hsa04145	Phagosome	1	P07996
hsa05205	Proteoglycans in cancer	1	P07996
hsa04514	Cell adhesion molecules (CAMs)	1	P16109

### PPI network

Studying the interactions between proteins and the networks formed by these proteins is important for revealing protein functions. Thus, we performed PPI network analysis of differential proteins. Combined_score indicates the support of protein interactions in the database, the larger the combined_score the stronger the interactions between the two proteins, and the screening threshold is 0.7 in this study. As shown in Fig. 4C, larger number of connections to other proteins indicated a greater degree of protein connectivity. In general, a greater degree of protein connectivity is associated with wider fluctuations in the entire system when the protein changes, indicating that the proteins play critical roles in maintaining system stability and balance and should be further evaluated.

### PRM analysis

Five proteins of interest were identified: Apolipoprotein A-I (ApoA1), Apolipoprotein A-II (ApoA2), Apolipoprotein C-III (ApoC3), Fibrinogen Gamma Chain (FGB), and fructose-bisphosphate Aldolase B (AldoB). These five proteins were also detected as significant signal molecules in KEGG pathway analysis. According to the PRM results, the five proteins displayed similar trends in LC-MS/MS analysis, supporting the reliability and credibility of the proteomics results (Table 4).

**Table 4:** Relatively quantitative analysis of target protein.

Protein Name	Average_control group	Average_refractory group	Ratio_refractory/ Control	p-value
ApoA1	0.7611	0.4643	0.61004723	0.09230385
ApoA2	2.5884	5.4602	2.10951153	0.05922255
ApoC3	0.6445	1.6855	2.61527828	0.00031998
FGB	0.0122	0.0202	1.65819646	0.30593333
AldoB	0.0564	0.1439	2.54897074	0.08950317

## Discussion

This is the first quantitative proteomics study of refractory BPPV among all types of BPPV. We identified key proteins potentially involved in the mechanism of refractory BPPV and biomarkers of refractory BPPV. Proteomics was first proposed by Wilkins in 1994.^20^ Compared with the traditional single gene or protein research, proteomics technology shows higher reliability in disease diagnosis and more accurately reflects changes related to some biological processes.^21^ TMT combined with LC-MS/MS is a novel technology for accurately and instantaneously comparing several samples for protein- and peptide-based labeling quantification.^22^ Proteomics analysis may be limited by false-positive results. Thus, the results must be confirmed in further research. In this study, we performed quantitative PRM analysis, which has been widely applied to assess many proteins.^23^

In this study, we identified 57 differentially expressed proteins. GO and KEGG pathway analysis revealed cholesterol metabolism, platelet activation, PPAR signaling pathway, and p53 signaling pathway as involved in refractory BPPV.

A study showed that the lipid profiles were higher in individuals with BPPV than in controls.^24^ von Brevern’s cross-sectional study indicated that hyperlipidemia, hypertension, and stroke were independently related to BPPV.^25^ These findings agree with those of another study which showed that the prevalence of hyperlipidemia, hypertension, coronary artery disease, and diabetes mellitus was higher in patients with BPPV than in controls.^26^ Based on our results, cholesterol metabolism and platelet activation likely participate in the occurrence and development of refractory BPPV.

The PPAR signaling pathway contains three important receptors. Peroxisome proliferator-activated receptors (PPAR-α, PPAR-β/δ, and PPAR-γ) are members of the nuclear receptor superfamily and play important roles in glucose and lipid metabolism. PPAR is considered as an important therapeutic target for hypertension, inflammation, and atherosclerosis.^27^ Hypertension and diabetes were reported as important risk factors for BPPV recurrence. This may be because of the decrease in blood flow in the labyrinth of the inner ear caused by hypertension and vascular diseases, resulting in displacement of the otolith and eventually the development of BPPV.^28^ Some studies proposed that histopathological changes of microangiopathy occurred in patients with chronically hyperglycemic diabetes mellitus. Because the inner ear accepts vascularization through the terminal branch, a decreased blood supply to the inner ear may damage vestibular function.^29^ A study showed that PPAR-γ and p53 expression levels were altered after rescue of BPPV, thereby attenuating oxidative stress in subjects with BPPV.^30^ Thus, the PPAR signaling pathway and p53 may also play important roles in refractory BPPV.

A total of five proteins were selected for PRM. Apolipoproteins are the main members of the plasma lipoproteins and have been demonstrated to play a key role in lipid metabolism.^31^ Both ApoA1 and ApoA2, which are critical components in the formation of high-density lipoprotein, are responsible for transporting cholesterol to the liver.^32^ ApoC3 plays a crucial role in the metabolism of triglycerides and triglyceride-rich lipoproteins. However, overexpression of ApoC3 is associated with hypertriglyceridemia.^33^ We found ApoA1 and ApoA2 were significantly changed in refractory BPPV. Furthermore, a lower level of ApoA1 and a higher level of ApoA2 and ApoC3 indicates the possibility of refractory BPPV disease related to cholesterol metabolism, indicating their roles as independent risk factors. Simultaneously, Ma et. al found that ApoA1 was decreased and ApoA2 was increased in Alzheimer's disease, which is strongly linked to oxidative stress.^34^ Many previous studies showed that oxidative stress plays an essential role in BPPV.^30^^,^^35^^,^^36^ It is worth noting that ApoA1 level is also downregulated in plasma from Meniere’s disease patients,^17^ which indicates a relationship between lipid metabolism and vestibular dysfunction. Therefore, a lower level of ApoA1 and higher level of ApoA2 may represent oxidative stress in patients with BPPV.

Aldolase B (AldoB) is the key enzyme in the conversion of fructose to methylglyoxal in vascular tissues.^37^ Vascular remodeling and development of hypertension are largely mediated by upregulation of AldoB. Fructose-upregulated AldoB expression promotes the conversion of fructose to methylglyoxal, which both contribute to vascular smooth muscle cell proliferation and vascular remodeling in hypertension.^38^ In our study, expression of AldoB was increased in patients. Thus, AldoB may mediate the occurrence of refractory BPPV through vascular factors. AldoB may also be a possible target for preventing and treating refractory BPPV. This finding provides an important direction for follow-up studies.

Fibrinogen levels have not been described in previous BPPV studies. We found that FGB and fibrinogen gamma chain were significantly increased in patient serum. FGB, fibrinogen gamma chain, and fibrinogen alpha chain can be polymerized to produce an insoluble fibrin matrix. Fibrin, as one of the core components of the thrombus, mainly functions in coagulation.^39^ FGB is involved in the physiological process of platelet activation. Platelet and fibrinogen play important roles in the beginning of acute cerebral ischemic stroke.^40^^,^^41^ It has been reported that a high level of plasma fibrinogen is a crucial risk factor for cerebral infarction.^42^^,^^43^ There is also evidence that fibrinogen alpha chain and gamma chain are upregulated in plasma from Meniere’s disease patients,^44^ which indicates that thrombogenesis might be one of the causes of vestibule dysfunction. In conclusion, our results suggest FGB, as one of the main components of fibrinogen, as a biomarker of refractory BPPV.

## Conclusion

This is the first study to use TMT and PRM-based quantitative proteomics to explore the mechanism of refractory BPPV. Proteomic results can provide essential information for understanding the pathological mechanism of refractory BPPV and developing diagnostic biomarkers. Additionally, proteomics can be useful in the development of new drugs. However, more extensive studies are needed to confirm the diagnostic value of these biomarkers. The precise molecular mechanisms of action also require verification through further functional studies.

## ORCID ID

Liang Xia: 0000-0003-1563-0083

Kexin Song: 0009-0003-5729-613X

Lili Xiao: 0000-0002-2019-8646

Yi Chen: 0009-0003-9679-5820

Hui Li: 0000-0002-2167-4931

Yanmei Feng: 0000-0003-4180-6564

## Funding

This study was supported by grants-in-aid from the 10.13039/501100001809National Natural Science Foundation of China (82371152 and 82171139) and the Joint research project of Pudong New Area Municipal Health Commission (PW2020D-9).

## Declaration of competing interest

The authors declare no conflicts of interest.
